# Treatment of cyclic vomiting syndrome with co-enzyme Q10 and amitriptyline, a retrospective study

**DOI:** 10.1186/1471-2377-10-10

**Published:** 2010-01-28

**Authors:** Richard G Boles, Mary R Lovett-Barr, Amy Preston, B UK Li, Kathleen Adams

**Affiliations:** 1 Division of Medical Genetics and the Saban Research Institute, Childrens Hospital Los Angeles, 4650 Sunset Blvd., Los Angeles, California, 90027 USA; 2 Department of Pediatrics, Keck School of Medicine at the University of Southern California, 4650 Sunset Blvd., Los Angeles, California, 90027 USA; 3 Cyclic Vomiting Syndrome Association, 2819 W. Highland Blvd., Milwaukee, Wisconsin, 53208 USA; 4 Division of Pediatric Gastroenterology, Medical College of Wisconsin, 9000 West Wisconsin Avenue, Milwaukee, Wisconsin 53226 USA

## Abstract

**Background:**

Cyclic vomiting syndrome (CVS), which is defined by recurrent stereotypical episodes of nausea and vomiting, is a relatively-common disabling condition that is associated with migraine headache and mitochondrial dysfunction. Co-enzyme Q10 (Co-Q) is a nutritional supplement that has demonstrated efficacy in pediatric and adult migraine. It is increasingly used in CVS despite the complete lack of studies to demonstrate its value in treatment

**Methods:**

Using an Internet-based survey filled out by subjects with CVS or their parents, the efficacy, tolerability and subject satisfaction in CVS prophylaxis were queried. Subjects taking Co-Q (22 subjects) were compared against those taking amitriptyline (162 subjects), which is the general standard-of-care.

**Results:**

Subjects/parents reported similar levels of efficacy for a variety of episode parameters (frequency, duration, number of emesis, nausea severity). There was a 50% reduction in at least one of those four parameters in 72% of subjects treated with amitriptyline and 68% of subjects treated Co-Q. However, while no side effects were reported on Co-Q, 50% of subjects on amitriptyline reported side effects (P = 5 × 10^-7^), resulting in 21% discontinuing treatment (P = 0.007). Subjects/parents considered the benefits to outweigh the risks of treatment in 47% of cases on amitriptyline and 77% of cases on Co-Q (P = 0.008).

**Conclusion:**

Our data suggest that the natural food supplement Co-Q is potentially efficacious and tolerable in the treatment of CVS, and should be considered as an option in CVS prophylaxis. Our data would likely be helpful in the design of a double-blind clinical trial.

## Background

Cyclic vomiting syndrome (CVS) is defined as recurrent identical episodes of nausea and vomiting, with the absence of these symptoms between episodes [1]. Prior to the advent of successful therapy, CVS was in most cases a disabling condition. Episodes of nausea and vomiting are generally severe and often require intravenous fluid therapy for dehydration. Frequent and prolonged school or work absences lead to academic or work disability. CVS is apparently common, being present in about 2% of Scottish [2] and Western Australian [3] school children.

CVS is generally believed to be a variant of migraine based on overlapping symptoms such as headache, nausea, and photophobia. There is also frequent progression of CVS to migraine headache, and a very high prevalence of migraine among close relatives of CVS patients [4]. Furthermore, CVS and migraine respond to many of the same medications including amitriptyline, cyproheptadine, propranolol and triptans. Amitriptyline (Elavil^®^), a tricyclic "antidepressant" is the most widely prescribed prophylactic medication used for the treatment of CVS. Response rates vary from 52-73% in open-label [5,6] and subject recall-based [4,7] studies in children and adults. In a recent consensus statement on management, amitriptyline was recommended as the first-line treatment choice for CVS prophylaxis in patients age 5 years and older [1].

Recently, the use of co-enzyme Q10 (Co-Q), also known as ubiquinone, has been gaining in popularity among CVS patient groups. Co-Q is a commonly-used dietary supplement that is widely available in retail settings. Co-Q is a naturally-occurring steroid-derived hydrophobic compound that is present in all organisms and essentially all food sources. Co-Q serves as the electron shuttle between complexes 1 or 2 and complex 3 of the mitochondrial respiratory chain [8]. It is thus essential for energy metabolism. Humans can manufacture this compound but often do so in inadequate quantities, with the remainder obtained from the diet. Physicians and other health care providers are increasingly recommending Co-Q for the treatment of a wide variety of conditions, including migraine [9], heart failure [10], statin treatment [11], neurodegenerative conditions [12], and primary inborn errors of mitochondrial energy metabolism [13].

Mitochondrial dysfunction is hypothesized to be a factor in the pathogenesis of both CVS and migraine headache based upon studies utilizing ^31^P-MRS [14] in migraine, and in both conditions by decreased respiratory complex enzymology [15-17], disease-associated mitochondrial DNA (mtDNA) sequence variants [18-23], and preferential maternal inheritance [4,24-27]. mtDNA is exclusively inherited from the mother. Three reasons prompted us to study whether Co-Q has efficacy in CVS prophylaxis: 1) demonstrated efficacy of Co-Q in migraine prophylaxis; 2) its increasingly popular usage in CVS; and 3) the association of both conditions with mitochondrial dysfunction.

In this retrospective study, we compare the efficacy, tolerability and patient satisfaction of Co-Q with the current standard-of-care in CVS therapy: amitriptyline.

## Methods

The study was advertised by the Cyclic Vomiting Syndrome Association (CVSA), a patient/family and professional led organization over a one-year period. Recruitment was conducted by means of newsletters, the CVSA website, e-mails to members and associated physicians, and by word of mouth. Individuals given a diagnosis of CVS by a health care professional, as attested to by the subject/parent by survey, were invited to participate in this study by completing an approximately 30-minute Internet-based survey (Survey Monkey). There was the option of completing the identical survey on paper. Subjects remained anonymous, and all aspects of this study were approved by the Childrens Hospital Los Angeles Institutional Review Board.

Efficacy was queried in terms of four different vomiting episode-based parameters: episode frequency, episode duration, number of emeses, and nausea severity. A reduction of at least 50% in each parameter was scored as positive and any lesser response as negative. The compound measure of "episode improvement" was scored as positive if at least one of the above four parameters were scored as positive. The data was reviewed in terms of efficacy and tolerability on low (<0.5 mg/kg/day), medium, and high (≥1.0 mg/kg/day) dosage amitriptyline, and for low and high (≥10 mg/kg/day or ≥ 300 mg in a patient ≥ 30 kg) dosage Co-Q. The data were also reviewed in terms of the child (<12 years), adolescent and adult (≥18 years) onset of stereotypical vomiting episodes. However, there were insufficient numbers of adolescent-onset subjects in order to fully characterize this group, and thus child- and adolescent-onset data were combined in order to create a "pediatric-onset" group. Chi square or Fisher exact tests were used as appropriate. Statistics were performed by WinSTAT Statistics for Windows, Kalmia Co. Inc., Cambridge, MA, and by custom-made software.

## Results

A total of 385 subjects completed the survey, of which 18 were excluded for the absence of a health professional-assigned diagnosis, and 13 for failure to meet both the Rome III [28] and NASPGHAN [1] diagnostic criteria for CVS based upon survey responses. Among the 347 subjects remaining, 277 and 82 reported experience with amitriptyline and with Co-Q, respectively. The final tally was 249 subjects for amitriptyline and 32 subjects for Co-Q after subtraction of 22 subjects who had started on both compounds at the same time, and 63 subjects that had started on Co-Q and/or amitriptyline and L-carnitine together. In both groups, 14 subjects were tried on both therapies at different times, and data on both are recorded.

Subjects reported treatment with a variety of different dosages of amitriptyline and Co-Q, yet no dose-response relationship or trend was evident. Furthermore, no relationship or trends were noted in regards to the age of onset of vomiting episodes with either therapy. One potential exception was a possible trend in a reduction of episode frequency by Co-Q in pediatric-onset (<18 years; 10/17) but not in adult-onset (1/5) CVS (P = 0.15). Thus, data for all dosages and ages were combined for statistical analyses.

In the compound measure of episode improvement, there was no difference reported between the therapies, with amitriptyline scoring positive in 72% and Co-Q in 68% (odds ratio = 1.2, 95% confidence interval 0.5-3.0). Furthermore, similar rates of response with both therapies were noted with all four episode parameters (Table 1).

**Table 1:** Efficacy, tolerability and patient satisfaction of co-enzyme Q10 and amitriptyline in the treatment of cyclic vomiting syndrome

	Amitriptyline			Co-enzyme Q10		
	**Pediatric Onset**^**1 **^**<18 y**	**Adult Onset ≥18 y**	**All Subjects**	**Pediatric Onset**^**1 **^**<18 y**	**Adult Onset ≥18 y**	**All Subjects**

**Episode Frequency**^**2**^	62/113	26/49	**88/162**	10/17	1/5	**11/22**
	55%	53%	**54%**	59%	20%	**50%**

**Episode Duration**^**2**^	59/112	19/43	**78/155**	5/16	3/6	**8/22**
	53%	44%	**50%**	31%	50%	**36%**

**Number of Emesis**^**2**^	52/110	6/16	**70/154**	6/16	2/4	**8/20**
	47%	41%	**45%**	38%	50%	**40%**

**Nausea Severity**^**2**^	47/111	21/46	**68/157**	7/18	4/7	**11/25**
	42%	46%	**43%**	39%	57%	**40%**

**Episode Improvement**^**3**^	88/123	39/54	**127/177**	12/18	5/7	**17/25**
	72%	72%	**72%**	67%	71%	**68%**

**Side Effects Reported**	72/139	30/63	**102/202**	0/20	0/8	**0/28**
	52%	48%	**50%**	0%	0%	**0%**

**Discontinued Therapy Due to Side Effects**	29/137	13/61	**42/198**	0/20	0/8	**0/28**
	21%	21%	**21%**	0%	0%	**0%**

**Subject Statement of Risks V. Benefits**^**4**^	49/94	14/40	**63/134**	13/16	4/6	**17/22**
	52%	35%	**47%**	81%	67%	**77%**

**Subjects' Recommendation**^**5**^	65/103	23/39	**88/142**	12/19	4/6	**16/25**
	63%	59%	**62%**	63%	67%	**64%**

**Subject on Therapy at Time of Survey**	68/111	30/48	**98/159**	14/19	6/7	**20/26**
	61%	63%	**62%**	74%	86%	**77%**

^1^The number of adolescent-onset cases was small, so the data were combined with the child-onset data.

^2^Proportion reporting a 50% reduction in this parameter

^3^Proportion reporting a 50% reduction in at least one of the above four parameters

^4^"Do you believe that the improvement you receive of [inset treatment name], if any, justified the side effects that you experienced?" - proportion answering as "yes, strongly" or "yes", as opposed to "sort of equal/not sure", "no" or "no, strongly".

^5^"Would you recommend [inset treatment name] to a friend or relative with CVS?" -- proportion answering as per ^4^above.

Side effects were frequently reported with amitriptyline (50%), and not recorded with Co-Q (P = 5 × 10^-7^) (Table 1). Among all subjects treated with amitriptyline, 21% discontinued (42/198) the drug because of side effects. None of the 28 patients stopped Co-Q because of side effects (P = 0.007). Queried as to whether the efficacy justified the side effects (Table 1), those taking Co-Q reported a significantly higher patient satisfaction than did those taking amitriptyline (77% responding as positive vs. 47%, P = 0.008, odds ratio = 3.6, 95% confidence interval = 1.2-10). However, when asked if they would recommend the agent to a relative or friend, the difference vanishes (positive responses: amitriptyline 62%, Co-Q 64%). Overall, there was a potential trend that subjects were more likely to remain on Co-Q versus on amitriptyline at the time of the survey (77% v. 62%, odds ratio 2.0, 95% confidence interval 0.75-5.2).

## Discussion

CVS generally results in severe discomfort, dehydration and disability, and despite prophylactic therapy substantial numbers of patients continue to suffer from repeated vomiting episodes. Our result of 72% efficacy (defined by compound measure) in those taking prophylactic amitriptyline agrees with the 52 to 73% efficacy rates reported in four previous studies [4-7]. This result also supports the overall validity of our on-line survey methodology. Our data is also consistent with our own clinical experience, in that overall efficacy is good (72%), yet side effects are frequent (50%), and often result in discontinuation of treatment (21%). Overall, approximately half (47%) of 134 subjects taking amitriptyline reported that the benefits outweighed the side effects of treatment. Furthermore, our data regarding the preventative treatment of CVS with Co-Q is consistent with our clinical experience, in that overall efficacy is good (69%), with side effects being rare (0/22). Three-quarters (77%) of subjects taking Co-Q reported that the benefits outweighed the side effects, a significantly higher portion than that with amitriptyline.

There are substantial child versus adult-related differences in clinical CVS, such as longer and more frequent episodes and more severe inter-episodic nausea among adults [29]. Furthermore, a strong association with the 16519T and 3010A mtDNA polymorphisms was noted in child-onset (< age 12 years), but not in adult-onset (≥18 years) cases [30]. Although the numbers of subjects in some categories were small, overall efficacy rates for both amitriptyline (70% v. 72%) and Co-Q (71% v. 71%) were very similar among subjects with child (<12 years) and adult (≥18 years) onset, respectively. Thus, our data do not suggest that the choice of either treatment should differ between pediatric or adult patients.

The limitations of this study are the subjective nature of retrospective subject/parent-completed questionnaires, the lack of validation data for our questionnaire, the lack of a proof of a physician-confirmed diagnosis of CVS, the self-selection bias in Internet questionnaire studies, the relatively small number of subjects reporting treatment with Co-Q, and the wide range of treatment durations, dosages, and in the case of Co-Q, brands and preparations (gel capsules, liquids, tablets, etc.) utilized. Interestingly, both amitriptyline and Co-Q seem to affect different parameters in different people, including episode frequency, episode duration, number of emeses, and severity of nausea. Neither therapy appears to affect the prodrome (data not shown). This information is important in the design of future prospective clinical trials in that complex end points for efficacy are needed. The failure to find a dose-response is expected as only the highest dosage attempted was queried, and thus the high-dose group may contain treatment failures that are not recorded as failures on lower doses.

The authors' clinical practice for treatment of CVS with Co-Q is 10 mg/kg/day divided bid, up to 200 mg bid (either in liquid or gel capsule formulation). It also includes obtaining a blood Co-Q level in the case of an inadequate response, and increasing the dose for blood levels less than 3 mg/L. Additional mitochondrial-targeted dietary supplements have also been used to prevent CVS attacks including L-carnitine, which has demonstrated efficacy in CVS in one small open-label study [31] (authors' practice: 100 mg/kg/day divided BID, up to one gram BID or TID), and riboflavin, which has demonstrated efficacy in migraine headache [32] (authors' practice: 100-400 mg/day, or one "B100" tablet a day). Anecdotally, in the authors' practice while some CVS patients respond well to cofactor therapy alone, many patients require drug therapy (generally amitriptyline) in addition. While this combination may have synergy based on anecdotal observations, the numbers of subjects in the current study are inadequate to address this question. This is another question for a prospective clinical trial.

## Conclusions

Our data suggest that the naturally-occurring food supplement Co-Q (ubiquinone) has potential therapeutic efficacy and excellent tolerability in the treatment of CVS. This data would likely be helpful in the design of a double-blind, prospective clinical trial, which is needed to definitively determine the appropriate role of Co-Q in CVS therapy. However, given the suggestion of efficacy, excellent tolerability, high patient satisfaction, and relatively low out-of-pocket costs (adult dose costs $15-20 U.S. per month from many discount stores), health care providers may want to consider it as an low-risk therapeutic option in this disabling condition.

## Competing interests

The authors declare that they have no competing interests.

## Authors' contributions

RGB conceived of the study, drafted the manuscript, and assisted in data analysis. MRLB performed most of the data analysis. All authors participated in designing the study (including the survey), recruiting subjects and physicians, and editing the manuscript.

## Pre-publication history

The pre-publication history for this paper can be accessed here:

http://www.biomedcentral.com/1471-2377/10/10/prepub
